# A new paradigm for *Aedes* spp. surveillance using gravid ovipositing sticky trap and NS1 antigen test kit

**DOI:** 10.1186/s13071-017-2091-y

**Published:** 2017-03-21

**Authors:** Sai Ming Lau, Tock H. Chua, Wan-Yussof Sulaiman, Sylvia Joanne, Yvonne Ai-Lian Lim, Shamala Devi Sekaran, Karuthan Chinna, Balan Venugopalan, Indra Vythilingam

**Affiliations:** 1 Selangor State Health Department, State Vector Borne Disease Control Unit, 29 Jalan Bayu Tinggi, 41200 Klang, Selangor Malaysia; 2 Department of Parasitology, Faculty of Medicine, University of Malaya, Lembah Pantai, 50603 Kuala Lumpur, Malaysia; 3 Department of Pathobiology and Medical Diagnostics, Faculty of Medicine and Health Sciences, Universiti Malaysia Sabah, Jalan UMS, 88400 Kota Kinabalu, Sabah Malaysia; 4 Department of Microbiology, Faculty of Medicine, University of Malaya, Lembah Pantai, 506030 Kuala Lumpur, Malaysia; 5 Julius Centre, Department of Social and Preventive Medicine, Faculty of Medicine, University of Malaya, Lembah Pantai, 50603 Kuala Lumpur, Malaysia; 6 Perak State Health Department, Jalan Panglima Bukit Gantung Wahab, 30590 Ipoh, Perak Malaysia

**Keywords:** GOS traps, Dengue NS1 kit, *Aedes* mosquito, Surveillance

## Abstract

**Background:**

Dengue remains a serious public health problem in Southeast Asia and has increased 37-fold in Malaysia compared to decades ago. New strategies are urgently needed for early detection and control of dengue epidemics.

**Methods:**

We conducted a two year study in a high human density dengue-endemic urban area in Selangor, where Gravid Ovipositing Sticky (GOS) traps were set up to capture adult *Aedes* spp. mosquitoes. All *Aedes* mosquitoes were tested using the NS1 dengue antigen test kit. All dengue cases from the study site notified to the State Health Department were recorded. Weekly microclimatic temperature, relative humidity (RH) and rainfall were monitored.

**Results:**

*Aedes aegypti* was the predominant mosquito (95.6%) caught in GOS traps and 23% (43/187 pools of 5 mosquitoes each) were found to be positive for dengue using the NS1 antigen kit. Confirmed cases of dengue were observed with a lag of one week after positive *Ae. aegypti* were detected. *Aedes aegypti* density as analysed by distributed lag non-linear models, will increase lag of 2–3 weeks for temperature increase from 28 to 30 °C; and lag of three weeks for increased rainfall.

**Conclusion:**

Proactive strategy is needed for dengue vector surveillance programme. One method would be to use the GOS trap which is simple to setup, cost effective (below USD 1 per trap) and environmental friendly (i.e. use recyclable plastic materials) to capture *Ae. aegypti* followed by a rapid method of detecting of dengue virus using the NS1 dengue antigen kit. Control measures should be initiated when positive mosquitoes are detected.

**Electronic supplementary material:**

The online version of this article (doi:10.1186/s13071-017-2091-y) contains supplementary material, which is available to authorized users.

## Background

Dengue was first reported in Malaysia in the early 1900s [1]. It became endemic in the 1960s and has emerged as a major public health problem in Malaysia from 1973 [2, 3] to the new millennium [4]. The two main vectors for dengue transmission in Malaysia are *Aedes aegypti* and *Aedes albopictus*, the former being the primary vector associated with dengue outbreaks [5, 6] and is also responsible for the transmission of chikungunya virus, yellow fever virus and Zika virus [7, 8]. Dengue has been a serious public health problem in tropical and subtropical countries [9, 10] and currently there is a 30-fold increase globally in incidence compared to 50 years ago [11]. In 2015, Zika virus has also become a huge public health problem in the Americas and may spread across the globe [12, 13].

The total number of dengue cases in Malaysia has increased 37-fold from 989 in 1973 [14] to 46,171 cases in 2010 [4]. The yearly rate of increase of reported cases rose from 47% between 2012–2013 to 62.1% between 2013–2014 [2012: 20,923 cases (35 deaths); 2013: 40,222 cases (92 deaths); 2014: 103,610 cases (215 deaths)] [15]. In 2015 there were 120,836 cases with 336 deaths [incidence rate, IR, of 396 cases per 100,000 population] where the State of Selangor contributed to the highest IR, of 1,076 cases per 100,000 population. Fatality rate in 2015 was 0.28% up from 0.2% in 2014. Selangor contributed the highest number of deaths (127) in 2015 (Report of the Communicable Disease control division of Ministry of Health Malaysia; Lau SM, personal communication).

Vector control has been the hallmark of the dengue control programmes in many countries in Southeast Asia [16] as anti-dengue drugs are not yet available. The most recent dengue vaccine is partly efficacious as it falls short of the levels of protection required for a standalone intervention [17]. House to house larval surveys, source reduction, larviciding, fogging, ULV which represent the old paradigms of dengue prevention and eradication are no longer practicable and need to be augmented by more targeted but less ambitious outbreak responses that focus on a few tools that might justify expense of deployment [18]. However, according to recent reports these tools (larval surveys, fogging, ULV) have not really been evaluated for their effectiveness in dengue control [19, 20]. These methods have been partially effective decades ago because *Aedes* house index ranged from 4.7 to 58.8% in the 1980’s [21] to 0.1 to 6.9% in the 1990s [22] and from 1.5 to 2% in recent years [15].

In the 1960s to 1980s the common breeding sites for *Aedes* in Malaysia were drums, earthenware jars, tires, bathtubs and ant traps [21–23]; however these have been replaced by discreet cryptic places which are more difficult for humans to locate [16]. Recent ecological studies in Penang, north Malaysia [24] showed increased diversity of containers, a shift to outdoor breeding opportunities and outdoor human daytime activities may increase exposure bites and transmission risks. Due to unplanned and increasing urbanisation since the mid-1970s, the control of the vectors has become more challenging [25, 26]. The lack of correlation between larval indices and dengue cases [27] and development of resistance by the *Aedes* to pyrethroids and temephos insecticides [28–31] pose a serious challenge to dengue control.

Of the control measures currently practised, targeted indoor residual spraying, thermal and ULV fogging are compromised by insecticide resistance and the transient nature of control [32]. Oviposition sites are often small, cryptic and difficult to locate, which makes effective larval control problematic. New paradigms for dengue control such as Release of Insects with Dominant Lethality (RIDL) and *Wolbachia* to control the *Aedes* population [33–36] require regulatory and lengthy approval in each country before they can be deployed to the field. In many countries studies are being conducted on the use of sticky traps to lure and trap gravid *Aedes* female adults [37–44]. These traps come in different designs and some contain insecticides or incorporate pyriproxyfen to kill the progeny [45, 46]. In a randomised control trial using BG sentinel trap, trapping slightly reduced the density of *Aedes* in the experimental area compared to the control area [47]. However, in a trial using CDC autocidal gravid trap (AGO) there was a significant reduction in adult *Ae. aegypti* between 53 to 70% in the intervention area compared to the control area [48].

Detecting dengue viral antigens from mosquitoes using antigen detection kits even by public health workers with minimum training has been reported [49–52]. In a previous study conducted in an urban area in peninsular Malaysia, we showed that the infected *Ae. aegypti* mosquito was obtained from sticky non-insecticidal traps before the first case was reported [53]. The trap actually targets gravid *Aedes* mosquitoes which can be infected. The Health Department staff involved in this study also found that it was easier to trap the adult mosquitoes than to carry out labour-intensive larval surveys.

The objective of this study was to evaluate the use of Gravid Ovipositing Sticky (GOS) trap and the NS1 testing system for surveillance of dengue virus transmission in urban Malaysia over a two year period.

## Methods

### Study site

The study was conducted in Mentari Court Apartments in the town of Petaling Jaya, State of Selangor (population 5.87 million), the most populated state in Malaysia. The details of the study site were previously described [53]. The study spanned over two years from November 2013 to December 2015 (week 47 in 2013 to week 47 in 2015). Mentari Court Apartments consist of seven blocks of 17 floors each with a total resident population of approximately 12,000 occupying 3,472 residential units.

### Trapping of mosquitoes using the GOS trap

A detailed description of the gravid mosquito ovipositing in sticky trap (GOS trap) was provided in [53]. The cost of each trap is less than USD 1. Briefly a total of 21 traps was deployed in each block (as determined from the pilot study [53]), three each on ground floor (GF), 3^rd^, 6^th^, 9^th^, 12^th^, 15^th^ and 17^th^ floor. The traps were set along the common corridors, 50–100 m apart and were placed near potted plants if available. All traps were filled with 7-day-old hay infusion water. The traps were checked weekly and the water changed during inspection. One ovitrap per floor was also set on the same floors as the GOS traps, mainly for the purpose of checking the presence of the *Aedes* mosquitoes.

Two teams consisting of two men each checked the traps weekly. Traps were inspected and those with mosquitoes on sticky surface were covered with a lid, placed inside a plastic container and brought back to the laboratory for further processing. If there were no mosquitoes the sticky sheets were changed monthly or as required if they were too dirty.

In the laboratory, the mosquitoes were identified morphologically to species. A pair of heat sterilised forceps was used to remove the mosquitoes from the sticky surface to prevent cross contamination. All the abdomens of the *Ae. aegypti* and *Ae. albopictus* were pooled (five per pool) for viral antigen detection tests which cost USD 4 per test. The head and thorax were individually stored in Eppendorf tubes at −80 °C until processed by RT-PCR to determine dengue virus serotypes.

### Detection of dengue viral antigen in pooled mosquitoes

The SD Bioline NS1 antigen kit (Standards Diagnostic, Gyeonggi-do, Republic of Korea) was used to test for dengue antigen in the pooled mosquito abdomens. In brief, 50 μl of PBS was added to the pooled abdomens and homogenised, the lysate was centrifuged briefly and the supernatant was added to the well of the test kit. After a lapse of 10–15 min, the reading was recorded. If two bands were present, the sample was considered positive. For negative samples, only the control band will appear. If the pooled abdomens were positive, the head and thorax of every individual of the pool were tested separately for dengue virus serotypes using multiplex RT-PCR. However, 25 head and thorax (from 5 pools of *Ae. aegypti*) were not subjected to RT-PCR due to misplacement of samples.

### RNA extraction and multiplex RT-PCR

Individual mosquitoes (head and thorax) were homogenised in pre-chilled Eppendorf tubes with 0.2 ml of growth medium (Minimum Essential Medium, MEM; Biowest, Missouri, USA). The homogenate was then centrifuged at 21,000× *g* for 15 min at 4 °C. RNA extraction was carried out using Cardo pathogen extraction kit (Qiagen, Hilden, Germany) and the kit’s protocol was strictly followed. The extracted samples were then subjected to one step multiplex RT-PCR using AccuPower RT-PCR PreMix (Bioneer, Seoul, South Korea) using the protocol of Yong et al. [54]. Briefly, this was a premix in a lyophilised form and was contained in 0.2 ml tubes. Thus, 15 μl of primer mix was added to each tube followed by 5 μl of the RNA template, vortexed and briefly spun. RT-PCR was performed in a Bio-RAD (Hercules, California, USA) PCR machine. The steps for this assay consisted of a 30-min RT step at 50 °C, 15 min of Taq polymerase activation at 95 °C, followed by 40 cycles of PCR at 95 °C denaturation for 30 s, 60 °C of annealing for 30 s and 72 °C extension for 1 min. Final extension was 72 °C for 10 min. Five μl of the PCR product was then analysed by gel electrophoresis.

### Dengue case data from Mentari Court Apartments

Data of serologically confirmed dengue cases (by NS1 or IgM/IgG) from the seven residential blocks were obtained from the Ministry of Health, Malaysia. It is mandatory for all hospitals and private practitioners to report cases to the Ministry of Health. The date of onset of case was used for all data analyses.

### Meteorological data

Data of weekly rainfall was obtained using rain guage RGR126 (Oregon Scientific Inc., Oregon, USA) in the study site. Maximum and minimum measures of temperature and humidity were obtained from the nearest meteorological station located five km from the study site.

### Statistical data analysis

All statistical analyses were done using weekly data and R programming language for statistical analysis (version 3.2.4) [55]. Preliminary simple linear and nonlinear correlation analysis indicated a lack of relationship between the environmental factors and total numbers of *Aedes* trapped, and between NS1-positive mosquito pools and dengue cases, due to lag effect. Subsequently we used the family of distributed lag non-linear models (DLNM), (*DLNM* package version 2.20 [56], which can simultaneously analyse non-linear factor-response dependencies and delayed effects, and provides an estimate of the overall effect in the presence of delayed contributions [57]. The effect of rainfall and temperature on the total number of *Aedes* trapped was investigated using the model: glm (Aedes ~ cb.temp + cb.rain, family = quasipoisson(), data); where cb = cross basis. For dengue cases, the model is: glm (case ~ cb.total_aegypti + cb.ns1positive + ns(time, 3) + woy, family = quasipoisson, data) where woy = week of the year. Both the *Ae. aegypti* trapped per week and cases per week at each trap floor were analysed separately by generalised linear mixed model (GLMM), using the block and floor as fixed factors, and the week as a random factor. Zero inflation and Poisson distribution were incorporated in the analysis. Differences in numbers of *Ae. aegypti* and cases between blocks and between floors were tested with Tukey’s contrasts at *P* = 0.05.

## Results

**Fig 1:**
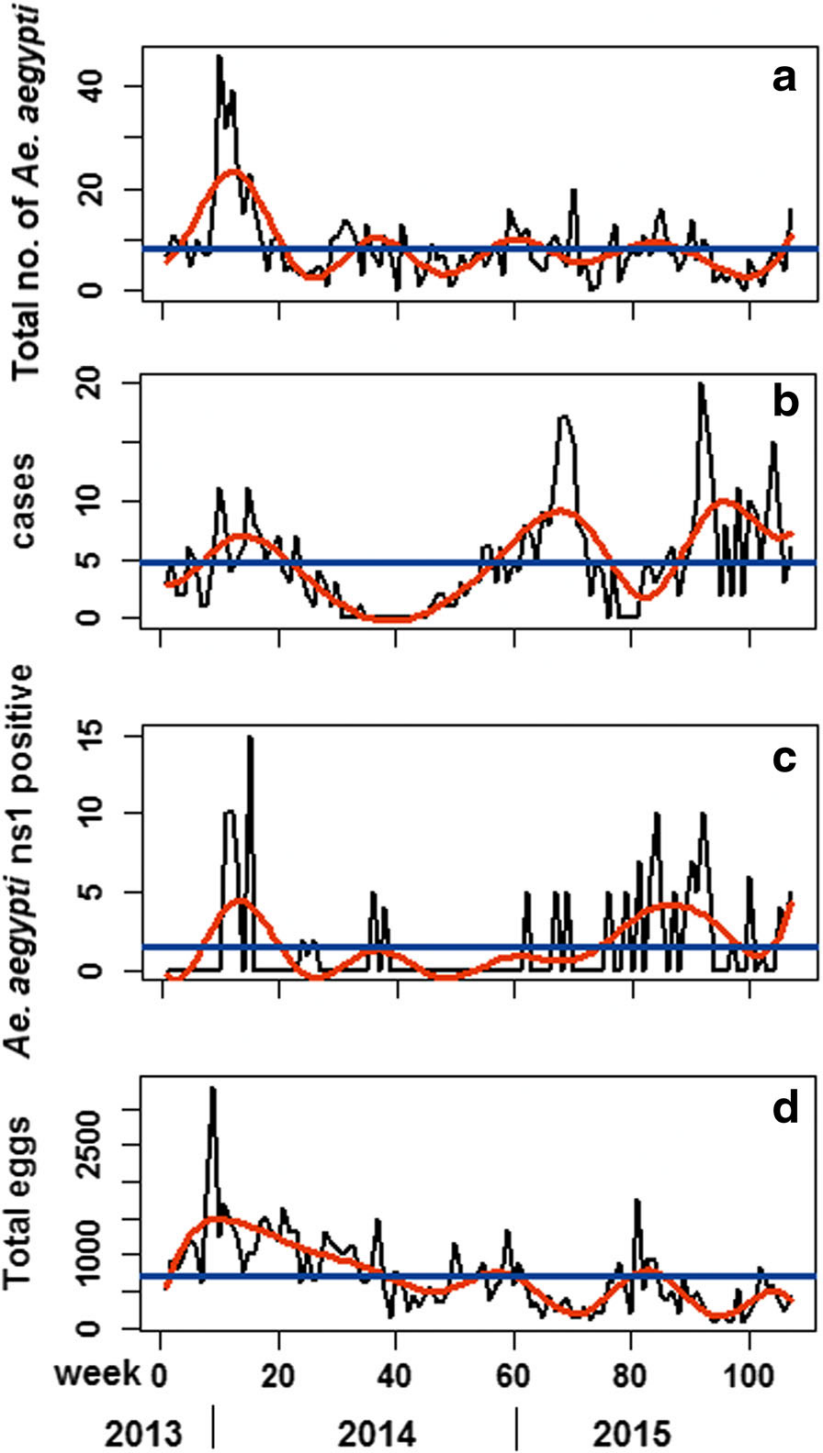
Time series of total number *Ae. aegypti* trapped per week (**a**), total number of dengue cases (**b**), number of *Ae. aegypti* found positive (**c**) and total number of eggs collected from the ovitraps (**d**) from November 2013 to December 2015, in Subang Jaya, Selangor, Malaysia. The solid red curve is a natural cubic smoothing spline, and the horizontal blue line indicates the overall mean value. Each total represents the sum of data from seven blocks with each block consisting of 21 traps

The study site was predominantly an *Ae. aegypti* (95.6%) area where 840 female (85%) and 148 male (15%) *Ae. aegypti* were caught compared to 37 female (80%) and nine male (20%) *Ae. albopictus.* The total number of *Ae. aegypti* trapped per week was highest in Jan 2014, thereafter the density followed a regular six-monthly pattern of higher numbers indicated by the spline graph, e.g. in June-July 2014, January and June-July 2015, rising again towards end of 2015 (Fig. 1a). As for number of cases, there were three peaks in: January 2014, March and August-September 2015 (Fig. 1b). The number of NS1 mosquito pools found positive followed the trend of the total number of trapped *Ae. aegypti* (Fig. 1c). The number of eggs collected followed the same pattern as the total number of *Aedes*, but the peaks appeared to decrease with time (Fig. 1d).

**Fig 2:**
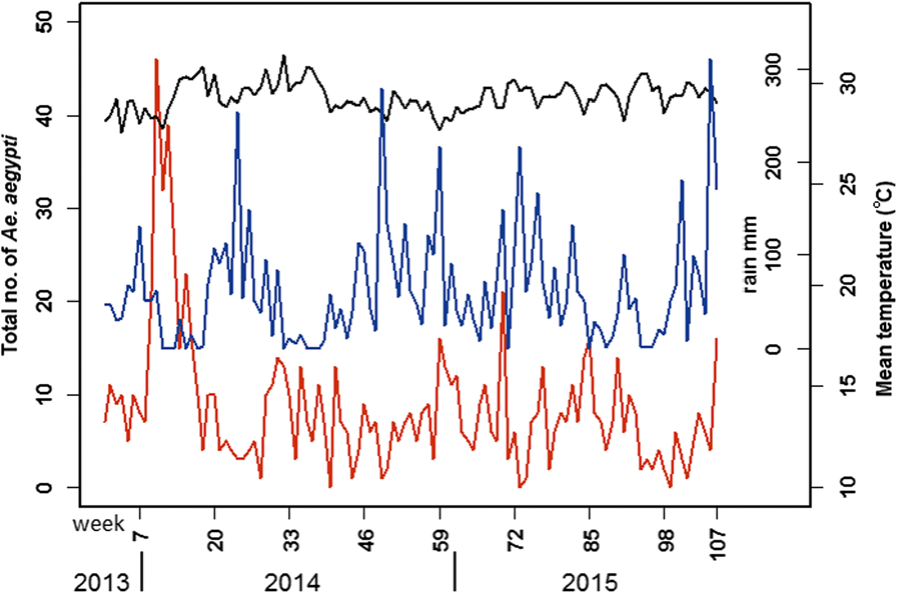
Plot of rainfall, mean temperature and total *Aedes aegypti* trapped per week in relation to time. *Key*: *red*, *Ae. aegypti* trapped; *blue*, rain; *black*, temperature (°C)

**Fig. 3:**
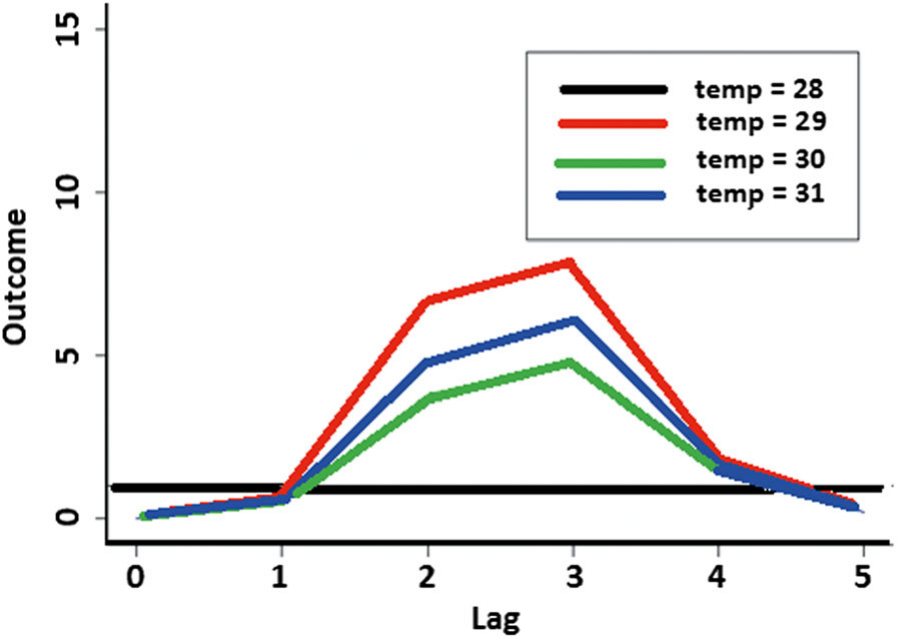
Lag-response curves of temperatures on weekly total numbers of *Aedes aegypti* trapped, with reference levels at 28 °C

**Fig. 4:**
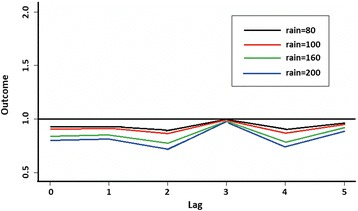
Lag-response curves of weekly rainfall on total numbers of *Aedes aegypti* trapped, with reference levels at 20 mm rainfall (line at 1.0)

The weekly mean temperature fluctuated within a narrow range between 27.6–31 °C (Fig. 2), and there was no discernible trend in the relationship between temperature and total number of trapped *Aedes*. However rainfall appears to have some relationship, albeit lagged. The plot of lag-response curves (Fig. 3) for different temperatures indicated that the number of trapped *Aedes* will be higher at 2–3-week lag if the temperature increased from 28 to 30 °C. Rainfall appeared to have a negative direct effect on the number of trapped *Aedes*, but positive effect was observed after the third week (Fig. 4), indicating *Aedes* number will be higher by a 3-week lag.

Significant difference was detected in the number of trapped *Ae. aegypti* between block B and E (Table 1). Similarly, *Ae. aegypti* was trapped more at ground level than any other floor, while 3^rd^ and 17^th^ floors were not different (Additional file 1: Figure S1).

**Table 1:** Mean number of *Ae. aegypti* trapped per week for each block and each floor as predicted by generalised linear mixed model

Block	No. of *Ae. aegypti* trapped per week	Floor	No. of *Ae. aegypti* trapped per week
A	0.3011 ^a^	Ground floor	0.8545^a^
B	0.2133 ^ab^	3^rd^ floor	0.3529^b^
C	0.2530 ^a^	6^th^ floor	0.2969^bc^
D	0.2531 ^a^	9^th^ floor	0.1697^c^
E	0.4021 ^ac^	12^th^ floor	0.2678^c^
F	0.3586 ^a^	15^th^ floor	0.2008^c^
G	0.2787 ^a^	17^th^ floor	0.3229^b^

Different letters along a column indicate the means are different significantly at *P* < 0.05 as tested by Tukey’s test

There was no difference in number of dengue cases reported during the study period between the floors, with values ranging 24 (floor 8) to 37 (floor 3). Interesting enough, floor 17 had 32 cases (Additional file 2: Figure S2). However, there was a significant difference between the blocks, with block E and G having the highest number of cases (Table 2; Additional file 3: Figure S3).

**Table 2:** Generalised linear mixed model fitting of the dengue cases data for 2013–2015

Block	Total cases	Predicted mean per week for block
A	59	0.725^a^
B	53	0.621^a^
C	60	0.728^ab^
D	45	0.536^a^
E	118	1.380^b^
F	65	0.778^ab^
G	109	1.294^b^

The model used is of the form “glmm < −glmmadmb (cases ~ block + floor + (1|year), zero Inflation = T, data = data, family = Poisson)”. Akaike information criterion (AIC) = 1,019.652. Block means with different superscript letters indicate they are significantly different at *P* < 0.05

**Fig. 5:**
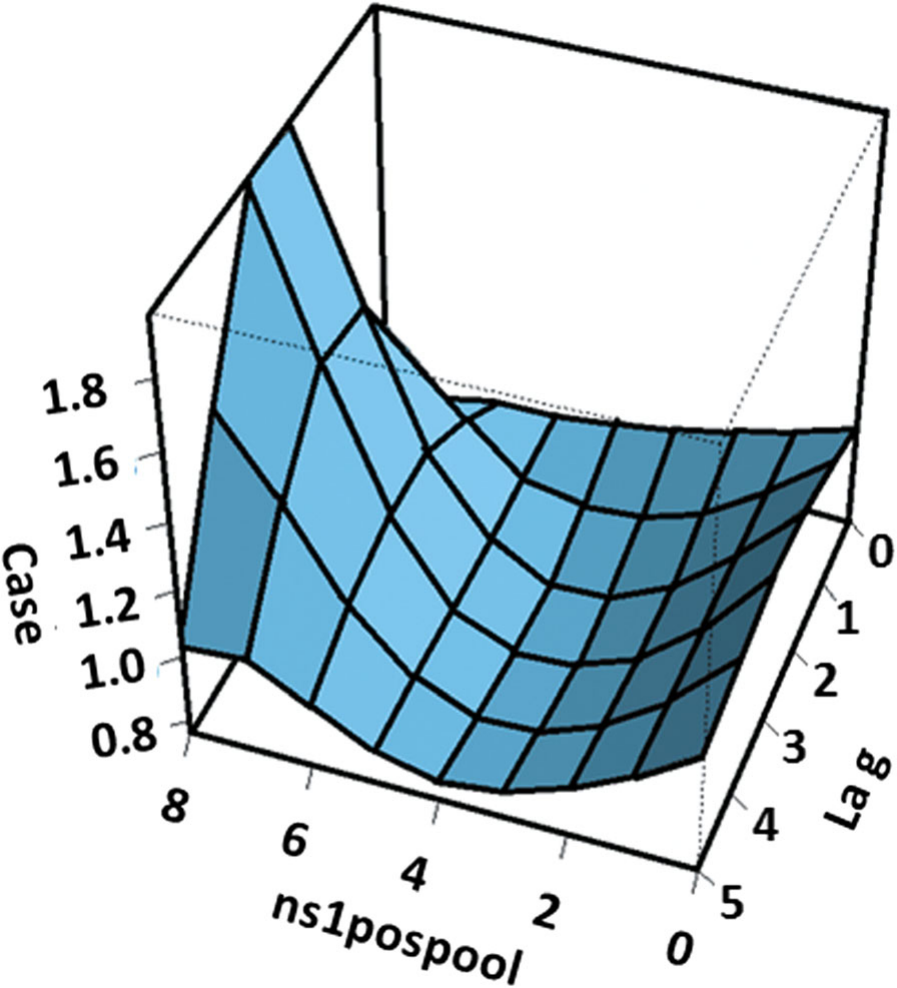
Three-dimensional plot of cases along NS1-positive mosquitoes and lags, with reference at none NS1-positive detected

**Fig. 6:**
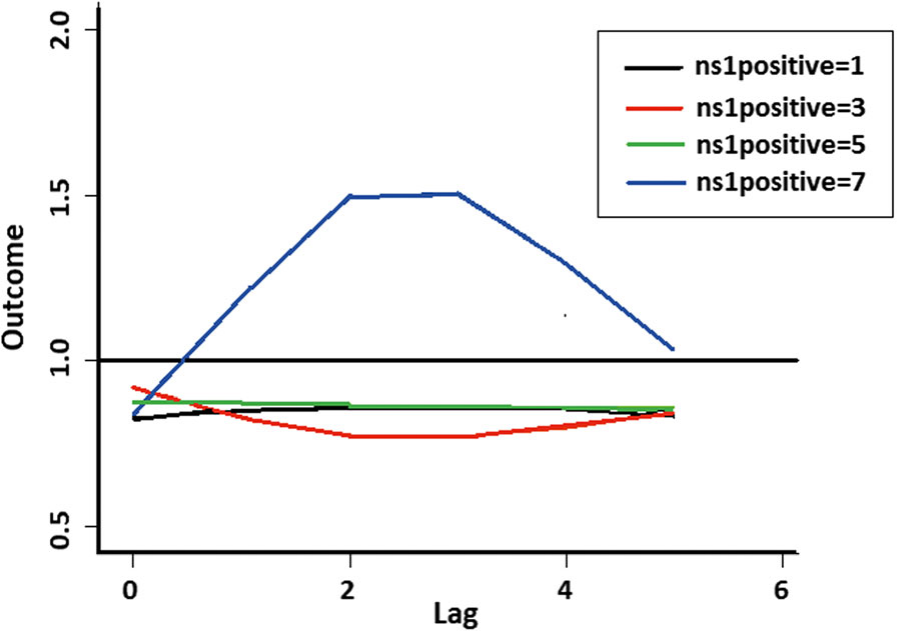
Plot of lag-response curves for different NS1-positive mosquitoes on dengue cases with reference line in NS1 positive (line at 1.0)

The relationship of number of dengue cases with both the number of NS1-positive mosquito pools and lag is depicted in Fig. 5. Cases occurred after a lag of one week after NS1-positive mosquito pool was detected, but peaked at 2 weeks lag. The plot of lag-response curves (Fig. 6) for different numbers of NS1-positive mosquito pools indicates that dengue cases will be highest at 2–3 weeks lag.

Forty-three pools of *Ae. aegypti* were positive by NS1 giving a minimum infection rate (MIR) of 51.2 per 1,000 (Additional file 4: Table S1). Only three *Ae. albopictus* pools were positive by NS1 but none of the heads and thoraces were positive by RT-PCR. The RT-PCR results of the individual head and thorax for *Ae. aegypti* were as follows: DENV1: 3; DENV2: 1; DENV3: 27; DENV2/DENV3: 3; DENV1/DENV3: 1. Three pools of mosquito (head and thorax) were negative. This may be due to the fact that the virus was still only incubating in the midgut and had not been disseminated to the salivary glands, or due to degradation of RNA in the mosquitoes. Heads and thoraces of mosquitoes from four negative pools were tested and shown to be negative by RT-PCR (Additional file 4: Table S2).

## Discussion

The increasing burden of dengue in Selangor, Malaysia is daunting and signifies a growing challenge to public health officials. Presently two teams of the health department staff are only able to inspect 40 premises (larval surveys) per day which could be increased to 3,000 premises if they were to use the GOS trap.

The main control strategies for dengue have not changed since their inception in the 1970s. House-to-house *Aedes* larval surveys followed by source reduction and larviciding remain the main tools for dengue control [16] not only in Malaysia but also in most other countries in Southeast Asia [16]. It has already been documented that these methods are not effective but they are still being used [14, 35, 36].

Studies have shown that *Ae. aegypti* can pick up dengue virus when biting asymptomatic or oligosymptomatic subjects [58] resulting in silent transmission from humans to mosquitoes. This might explain why dengue epidemics are on the rise. The present study indicates that the detection of dengue-positive mosquito will give rise to dengue cases after a lag of one week. This lends credence to our hypothesis that one way forward for dengue surveillance is the use of GOS trap coupled with the use of NS1 antigen kit for the detection of the virus in mosquitoes. Sensitivity of NS1 antigen kit on mosquitoes containing the virus has been established to be high (95%) [49]. According to Sylvestre et al. [52] the NS1 antigen kit has higher sensitivity compared to the qRT-PCR and virus isolation on dried *Aedes* mosquitoes.

Although the sticky traps may be a good and cheap alternative to trap *Ae. aegypti*, their ability to suppress *Aedes* population is variable. In Brazil [47] no reduction in the *Aedes* population was detected in the treated areas while in Puerto Rico they managed to suppress the *Ae. aegypti* population [48]. However, a comparative study in parts of Brazil using various traps and comparing them to regular house surveys found that the traps produced better results compared to *Aedes* house index [59]. Thus it is more important in dengue-prone areas to test the mosquitoes for dengue virus and institute control measures when positive mosquitoes are obtained.

Previous studies have shown that ovitraps were useful indicators for the presence of *Aedes* mosquitoes [60–62], but the association between ovitraps and dengue cases has not been established. Since a single female *Ae. aegypti* is likely to deposit the eggs in several containers due to skip oviposition behaviour [63], the ovitrap index is not a useful indicator for surveillance. In our study, there was significant correlation between the number of eggs per ovitrap and the number of adults caught per trap (Additional file 5: Figure S4). However, ovitrapping might not be useful for a surveillance programme because it allows an infected mosquito to lay eggs as well as to continue infecting people. The advantage of the GOS trap is that it traps the gravid mosquito which can then be used for virus detection.

Our data indicate there was no difference between the floors in terms of number of cases, although there were more mosquitoes trapped in lower floors. *Aedes aegypti* was also found breeding in the water tanks on the roof top which could explain the higher number of *Ae. aegypti* on floor 17. Nevertheless, dengue infection can occur in any of the floors.

Several studies had been conducted to determine the correlation between climate changes, dengue cases and adult mosquito abundance in the Asia-Pacific region and in the Americas to provide proactive indicator for dengue surveillance [64–66] with varying results. In this study we also analysed the association between weather and *Ae. aegypti* abundance at micro- level to determine if it could be used as a surveillance tool for dengue control. We found that if the temperature increased from 28 to 30 °C, the abundance of *Ae. aegypti* will increase with a lag of two weeks; while after rainfall the increase will be with a lag of three weeks. The shorter lag could be due to higher human density, an environment conducive to mosquito breeding in the study area and global warming. In Bangkok the cases increased two months after a heavy rainfall [67], in Puerto Rico it was confirmed that in areas where rainfall was uniformly distributed there was no correlation between rainfall and *Aedes* dynamic [66], while in areas where rainfall was more seasonal there was strong correlation with *Aedes* density and dengue cases [63]. In Singapore the effects of weather (absolute humidity, temperature, rainfall, relative humidity, wind speed) on dengue cases from 2001 to 2009 showed that absolute humidity was the best predictor and indicator for dengue [65].

Taking all these factors into consideration it would be more cost-effective to setup the GOS traps and monitor the adult population for dengue virus. As suggested one way forward is a package of proactive measures that aim to prevent, diminish or eliminate dengue transmission [11]. The study in Thailand using RT-PCR to detect the dengue virus in mosquitoes also showed a positive association between infected *Ae. aegypti* and dengue-infected children [68]. That study demonstrated the occurrence of an infected mosquito prior to the reporting of the index case(s). It has been stated recently that dengue virus transmission varies from year to year and place to place making vector control interventions difficult [69], thus it is timely for new measures to be introduced for dengue control instead of relying on reactive tools. The GOS traps can at least be introduced in hot-spot areas where dengue outbreaks occur. This GOS trap can also be used in public places such as transportation hubs (train stations, bus stops, etc.) recreation areas and commercial areas as viral-positive *Aedes* have also been obtained from these areas (Lau SM, personal communication).

If this method is adopted, once the positive mosquito has been detected, health department teams could move into action and carry out control measures even before cases are reported. The GOS traps would eliminate gravid female mosquitoes including the infective individuals as they attempt to oviposit inside the traps. Vector control measures targeting the adult mosquitoes will potentially lower vectorial capacity of infected and incubating mosquitoes and reduce mosquito density below a threshold to prevent dengue outbreaks. At the same time people who fall ill will be aware that it might be dengue and may seek treatment early thus preventing mortality. This approach could be considered as a replacement for the laborious, difficult, time-inefficient, insensitive and costly house-to-house larval surveys [69].

## Conclusions

This study has shown that the use of GOS traps and NS1 kit represents one possible way forward to forewarn and reduce dengue outbreaks which are increasing yearly and projecting a global disease burden. For a start the strategy provides early warning system where swift action can be taken by public health workers to reduce dengue outbreaks. High dengue transmission rates across Southeast Asian countries with extensive diversity in population density, climate, and geology may be explained by the infectiousness of asymptomatic cases to *Ae. aegypti* [58]. The situation is exacerbated due to a long or delayed response time for fogging and ULV space spraying after a case has been reported. The response may be more efficient when timely vector control measures are implemented after the immediate detection of an infected mosquito from the GOS trap. This study has shown that dengue cases will occur after a lag of one week following the detection of a viral-positive mosquito. However, further research especially a randomised control trial should be carried out to evaluate the actual effectiveness of combination of GOS trapping and NS1 antigen testing before it can be integrated into a control programme.

## Additional files

Additional file 1: Figure S1. Percentage of female *Ae. aegypti* caught in each floor for all seven blocks. (TIF 67 kb)

Additional file 2: Figure S2. Number of dengue cases recorded during the study period (2013–2015) plotted according to the floor. (TIF 74 kb)

Additional file 3: Figure S3. Number of dengue cases recorded during the study period (2013–2015) plotted according to the block. (TIF 66 kb)

Additional file 4: Table S1. Total pools and number of mosquitoes positive by weeks using NS1 Rapid Test Kit. **Table S2.** Mosquito pools tested by NS1 and RT-PCR. (DOCX 15 kb)

Additional file 5: Figure S4. Relationship between the weekly numbers of *Ae. aegypti* caught per trap and number of eggs per ovitrap. There was a significant correlation between the number of eggs per ovitrap (mean 12.66, range 1.52–56.81) and the number of adults caught per trap (*r* = 0.41, *t* = 4.5, *df* = 105, *P* < 0.001). (TIF 1980 kb)

## Abbreviations

DLM: Distributed lag linear model
DNLM: Distributed lag non-linear models
GLMM: Generalised linear mixed model
GOS: Gravid ovipositing sticky
IgG: Immunoglobulin G
IgM: Immunoglobulin M
IR: Incidence rate
RT-PCR: Reverse transcriptase polymerase chain reaction
ULV: Ultra-low volume
