# Supplementary figures and images for: A new paradigm for Aedes spp. surveillance using gravid ovipositing sticky trap and NS1 antigen test kit
Source: Parasit Vectors. 2017 Mar 21;10:151. doi: 10.1186/s13071-017-2091-y (PMC5361725; doi:10.1186/s13071-017-2091-y)

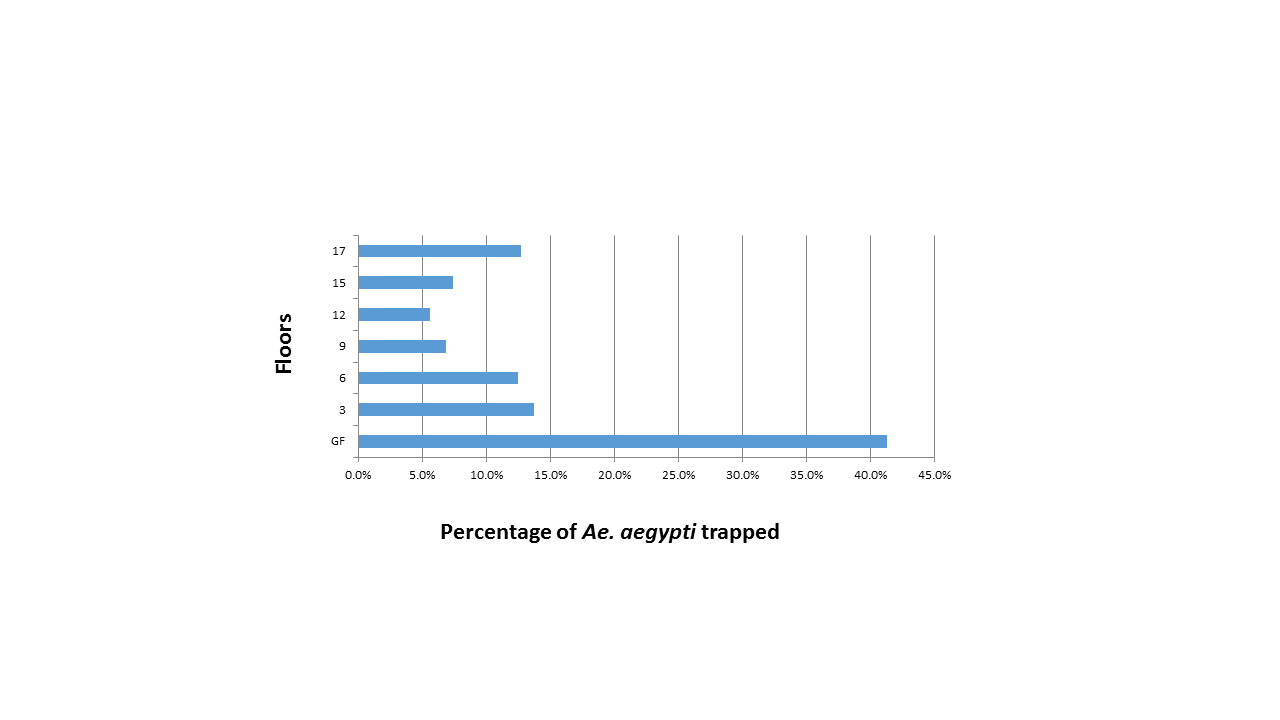

Supplement: Supplementary file 1 — Percentage of female Ae. aegypti caught in each floor for all seven blocks. (TIF 67 kb) [file 13071_2017_2091_MOESM1_ESM.tif]

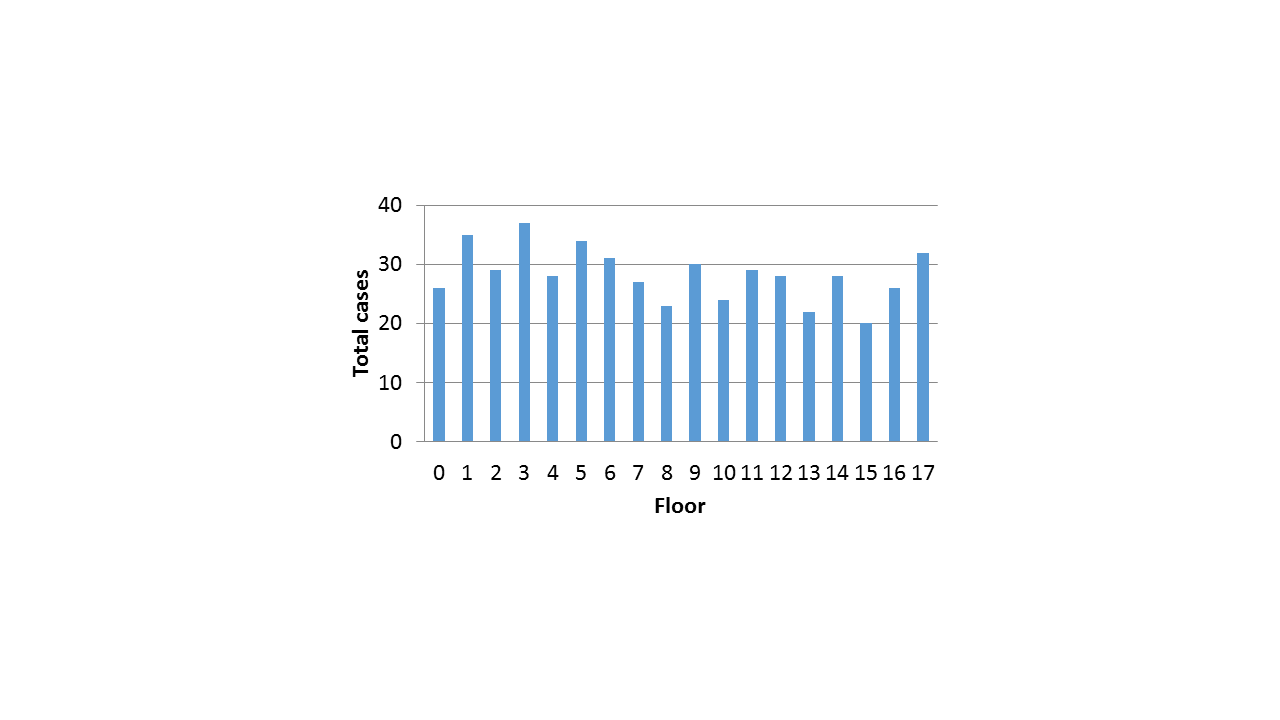

Supplement: Supplementary file 2 — Number of dengue cases recorded during the study period (2013–2015) plotted according to the floor. (TIF 74 kb) [file 13071_2017_2091_MOESM2_ESM.tif]

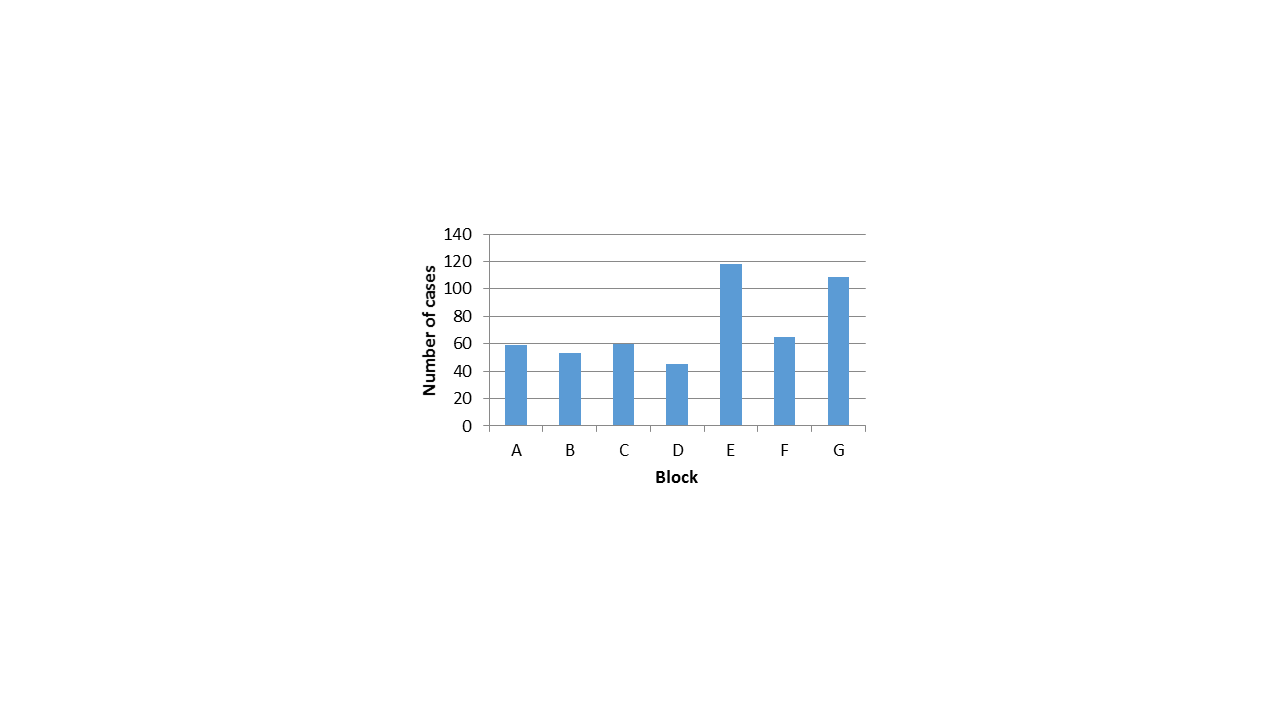

Supplement: Supplementary file 3 — Number of dengue cases recorded during the study period (2013–2015) plotted according to the block. (TIF 66 kb) [file 13071_2017_2091_MOESM3_ESM.tif]
