# Supplementary material for: A new paradigm for Aedes spp. surveillance using gravid ovipositing sticky trap and NS1 antigen test kit
Source: Parasit Vectors. 2017 Mar 21;10:151. doi: 10.1186/s13071-017-2091-y (PMC5361725; doi:10.1186/s13071-017-2091-y)
Supplement: Supplementary file 4 — Total pools and number of mosquitoes positive by weeks using NS1 Rapid Test Kit. Table S2. Mosquito pools tested by NS1 and RT-PCR. (DOCX 15 kb) [file 13071_2017_2091_MOESM4_ESM.docx]

| **Additional file 5. Table S1** Total pools and number of mosquitoes positive by weeks using NS1 Rapid Test Kit. | | | | | | | | | |
| --- | --- | --- | --- | --- | --- | --- | --- | --- | --- |
|  |  |  |  |  |  |  |  |  |  |
| Year | Week | NS1 Antigen Test | | | | | | | |
|  |  | *Aedes aegypti* | | |  | *Aedes albopictus* | | | |
|  |  | Total pools (mosquitoes tested) | Total pools positive (number of mosquitoes) | % Positive pools |  | Total pools (mosquitoes tested) | Total pool (number of mosquitoes ) | % Positive |  |
| 2013 | wk47 - wk53  (7 week) | 9  (46) | 0 (0) | 0.00 |  | 1(1) | 0 pool (0 mosquito) | 0.00 |  |
| 2014 | wk1 - wk53  (53 week) | 105 (475) | 13 (56) | 12.38 |  | 12 (30) | 2 (2) | 16.67 |  |
| 2015 | wk1 - wk47  (47 week) | 73 (319) | 30 (135) | 41.10 |  | 2 (6) | 1 (1) | 50 |  |
|  | Total: | 187 (840) | 43 (191) | 22.99 |  | 15 (37) | 3 (3) | 20 |  |

Additional file 5. Table S2 Mosquito pools tested by NS1 and RT-PCR

| Pool | Not tested | Positive in RT-PCR (at least one mosquito positive in the NS1 pool) | Negative in RT-PCR | Total |
| --- | --- | --- | --- | --- |
| Positive NS1 pool | 5 | **35** | 3 | 43 |
| Negative NS1 pool | 140 | 0 | 4 | 144 |
| Total | 145 | 35 | 7 | 187 |
